# A Narrative Review of Robotic Aquablation in Benign Prostatic Hyperplasia Care: Where We Are Now

**DOI:** 10.7759/cureus.96677

**Published:** 2025-11-12

**Authors:** Mohammad Ekhlasur Rahman, Faisal Haque, Muhammad Rakib Hasan, Mahabub Hassan, Rezuana Tamanna

**Affiliations:** 1 Urology, Watford General Hospital, West Hertfordshire Teaching Hospitals NHS Trust, Watford, GBR; 2 Urology, Broomfield Hospital, Mid and South Essex NHS Foundation Trust, Chelmsford, GBR; 3 Urology, Epsom and St Helier University Hospitals NHS Trust, Sutton, GBR; 4 General Surgery, Craigavon Area Hospital, Northern Ireland Medical and Dental Training Agency, Portadown, IRL

**Keywords:** benign prostatic hyperplasia, bph, mis, prostate, robotic aquablation

## Abstract

Benign prostatic hyperplasia (BPH) is a condition causing lower urinary tract symptoms (LUTS) that impair men's health and quality of life for years. The well-established surgical treatments, like transurethral resection of the prostate (TURP), may pose risks of thermal injury and subsequent sexual dysfunction. This narrative review summarizes the current evidence for robotic Aquablation, a novel, image-guided therapy for BPH. Aquablation utilizes a heat-free, high-velocity saline jet, robotically controlled under real-time ultrasound, to precisely resect tissue while preserving crucial collagenous structures like the bladder neck and ejaculatory ducts, so this may preserve the functional outcomes. Clinical trials and prospective studies have consistently demonstrated that Aquablation provides significant and durable improvements in urinary outcomes, including International Prostate Symptom Score (IPSS) and peak flow rate (Qmax), comparable to TURP. Its primary advantage is the superior preservation of sexual function, with significantly lower rates of ejaculatory dysfunction compared to standard procedures. Furthermore, a more formal meta-analysis and systematic review incorporating newer studies is desired.

## Introduction and background

Benign prostate hyperplasia (BPH), also known as an enlarged prostate, is a widespread condition affecting men's urinary function and overall health and quality of life (QoL). BPH commonly presents with a spectrum of lower urinary tract symptoms (LUTS) across the storage and voiding domains, including urinary frequency, urgency, weak stream, and a sensation of incomplete emptying, which may progress to acute urinary retention and increase susceptibility to urinary tract infections [1-4]. 

In 2024, about 29-33% of men aged 65 and older had BPH, with an average annual incidence of roughly 600 (6%) per 10,000 men [5]. Most cases of BPH are initially managed non-surgically through active surveillance and medical therapy [6]. 

Surgery is recommended for LUTS caused by BPH when non-surgical treatments are ineffective or in specific cases like urinary retention [7]. For small- to moderate-sized glands, transurethral resection of the prostate (TURP) is considered the traditional, widely used operation and is recognized as the gold standard [6,8,9]. Newer methods involve minimally invasive surgical treatments like transurethral laser photovaporization of the prostate (PVP) and non-tissue removal techniques such as steam injection therapy (Rezum) and implants (UroLift). In contrast, enucleation techniques remain consistently suitable, and the patient's unique characteristics, along with the surgeon's expertise, should be considered and highlighted when evaluating surgical options [6,7]. 

Current surgical BPH treatments carry risks of thermal injury, leading to urinary incontinence, erectile dysfunction, and ejaculatory dysfunction. While minimally invasive techniques such as UroLift and Rezum exist, their applicability is limited to smaller prostates [10,11]. Aquablation (AquaBeam; PROCEPT BioRobotics, Redwood Shores, California, United States) is a heat-free, image-guided, robot-assisted waterjet (hydrodissection) technique that uses real-time ultrasound mapping to plan a conformal resection and a high-velocity saline stream to selectively ablate prostatic parenchyma while sparing surrounding collagenous structures, offering precise debulking of BPH tissue responsible for LUTS [12]. In this review, we aim to provide a comprehensive overview of the current evidence and practice of robotic Aquablation in the management of BPH, discuss its comparative effectiveness, and highlight its limitations, complications, and future directions.

## Review

Technological overview

High-pressure waterjet technology is initially a staple in industrial usage for the precision cutting of metal, ceramic, wood, and glass. This advanced technique has been successfully repurposed for the intricate dissection of parenchymal tissues in both animal and human models with notable precision. Its pioneering application in the medical field was in liver resection. Initially demonstrated in canine subjects, the technique was subsequently applied to human patients, enabling the selective dissection of liver parenchyma while meticulously preserving the integrity of vital structures such as bile ducts and blood vessels [13-15].

The adoption of this technology has been shown to be notably effective for both open and laparoscopic surgical resection across a diverse range of parenchymal organs, including the brain, kidneys, and lungs [16-20]. Recently, hydrodissection and waterjet technology, similar to that used in Aquablation, have become safe and effective methods for the transurethral resection of bladder tumors [20].

Ablation offers a safe and effective alternative to the traditional cutting of prostate adenoma in the management of BPH. Over the years, a variety of advanced techniques have been employed for prostatic ablation, including the use of various forms of lasers, which precisely target and remove prostatic tissue, and electrosurgical techniques, which utilize electrical energy to achieve similar therapeutic outcomes [21,22].

The Aquablation system provides a consistent, measurable, and standardized heat-free ablation for tissue, effectively widening the prostatic urethra between the bladder neck and peripheral sphincter. The Aquablation system operates in two phases: The first phase is the "cutting mode", where the surgeon adjusts settings like angle and depth and the system then uses a high-pressure (500-8000 PSI) waterjet of physiologic saline to cut and dissect the soft tissue as planned. The second phase is the "coagulation mode", which follows the resection; in this phase, the console switches to a low-pressure pump (5-15 PSI) and activates a 2-watt green light laser (532 nm) to allow for cauterization [23]. This innovative technology leverages real-time ultrasonic imaging, which not only facilitates the precise surgical planning and mapping of the prostate but also enables the controlled and complete ablation of prostatic tissue using a powerful, high-velocity saline stream. The initial assessment of Aquablation's safety and feasibility was conducted in a canine model, demonstrating its potential before human trials [12,24].

Description of the Intervention With the AquaBeam System

The surgical procedure is predominantly automated, requiring the patient to be under general anesthesia for the duration of the intervention [24,25]. The AquaBeam system comprises a console, a robotic handpiece, and a single-use probe. Access is gained transurethrally with a cystoscope; the obturator is removed, leaving the sheath at the bladder neck to serve as a working channel. The handpiece is advanced until its balloon lies in the bladder, inflated with 15 mL saline, and retracted to seal the neck, preventing antegrade flow. The articulating arm is locked to stabilize the device [12,24]. 

**Figure 1:**
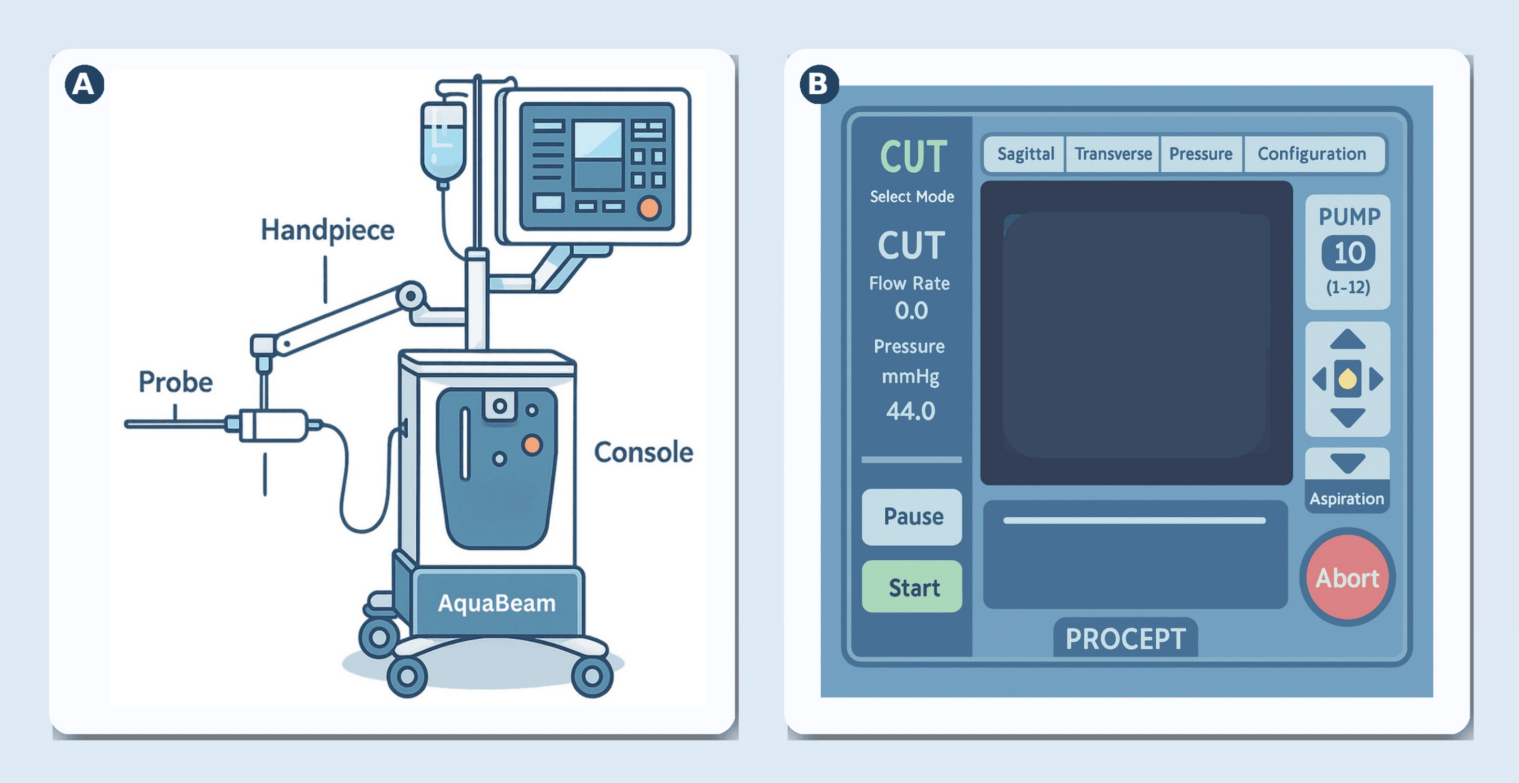
Aquablation (AquaBeam) system: components and console interface (A) Schematic of the Aquablation platform used to treat BPH. The mobile console houses the robotic drive and ultrasound-guided planning software. A sterile handpiece on an articulated arm couples to a disposable transurethral probe that delivers a high-velocity saline waterjet to resect adenomatous tissue without thermal energy. Resection is performed under real-time TRUS guidance with the surgeon defining the target resection map; the robot executes the planned passes to achieve conformal, standardized ablation. (B) Representative console user interface during the "CUT" mode. Ultrasound views (e.g., sagittal and transverse) are used for planning and intraoperative visualization; pump pressure/flow and aspiration are adjusted from the console; Start/Pause/Abort controls govern automated treatment passes, while the surgeon supervises and can modify parameters. The overall workflow (image-guided mapping → robotic, heat-free waterjet resection) reflects the system's design described in the initial clinical experience report. BPH: benign prostatic hyperplasia; TRUS: transrectal ultrasound The figure was created by the article authors as an illustration for the AquaBeam system with reference to [12,24,25] using Adobe Illustrator (Adobe Inc., San Jose, California, United States).

A bi-plane transrectal ultrasound (TRUS) probe on a stepper provides imaging; prostate dimensions are manually entered on the console to define the resection map. Treatment begins via foot pedal: the console powers a pump that emits a high-velocity saline jet at 90°, modulating flow, rotation, and longitudinal sweep of the probe to follow the plan and hydro-resect tissue to the mapped contour. Finally, a thin cautery layer (0.5-1 mm) using a 3-5 W laser is applied, the device is removed, and a Foley catheter is placed. Reported adverse events include postoperative pain, hematuria, urinary tract infections, urethral stricture disease, acute urinary retention, and one instance of blood transfusion (Figure 1) [24]. 

Efficacy, safety, and sexual function preservation after Aquablation

The 2019 amendment to the American Urological Association (AUA) guidelines represents a significant update, officially incorporating Aquablation as a viable treatment alternative for benign prostatic enlargement (BPE). This inclusion specifically targets cases involving small- to medium-sized prostates, expanding the therapeutic options available to patients and clinicians [26]. Also, we should note that the 2020 European Association of Urology guidelines highlighted Aquablation as a viable alternative to TURP [27]. This recommendation highlights the growing acceptance and efficacy of Aquablation in the management of benign prostatic hyperplasia (BPH) within this specific prostate volume range.

Research has demonstrated that Aquablation is both safe and effective, requiring only a relatively short learning period for medical professionals to become proficient while quickly demonstrating its safety and efficacy [28,29]. Beyond the functional results, a number of post hoc and subgroup analyses have been published from these clinical trials, including the US cohort from the Waterjet Ablation Therapy for Endoscopic Resection of Prostate Tissue (WATER) studies. Twelve months after the phase II trial, patients experienced marked relief in urinary symptoms: International Prostate Symptom Score (IPSS) and IPSS-QoL scores fell significantly, and urinary flow (Qmax) increased (all p<0.01). The mean post-void residual dropped by 89 mL. Sexual function was maintained; the ejaculatory and orgasmic domains showed no decline, overall International Index of Erectile Function (IIEF)-15 trended upward, and intercourse satisfaction (items 6-8) improved significantly (p<0.01) [30].

Zorn et al. reported durable three-year outcomes after Aquablation: peak urinary flow nearly doubled to 18.5 mL/s, post-void residual fell markedly to 51 mL, and serum prostate-specific antigen (PSA) declined (7.1 to 5.0). By year 3, only 6% of patients required BPH medication, and 3% underwent additional surgery for LUTS. Notably, baseline symptom severity did not modify treatment effectiveness. Together, these findings support Aquablation as a safe, effective, and durable treatment option for BPH [31].

A retrospective single-center study from Taiwan (n=18; March-July 2024) evaluated Aquablation outcomes for BPH/LUTS. Significant improvements were observed across several metrics (all p≤0.03): IPSS decreased from 17.9 to 6.2, QoL from 3.9 to 1.6, and post-void residual from 118 to 34 mL. Urine flow rates also improved, with Qmax rising from 10.9 to 27.4 mL/s and Qmean from 4.8 to 11.3 mL/s. The mean hemoglobin drop was 1.76 g/dL, with one patient requiring a transfusion. Postoperative bleeding necessitated reoperation in three patients, though no sexual dysfunction was reported at short-term follow-up. The study concluded that Aquablation is a promising alternative offering substantial functional gains, emphasizing the need for larger, longer-term studies in Taiwan [10].

Bach et al. conducted OPEN WATER, a prospective, multicenter, single-arm "all-comers" study evaluating Aquablation in 178 men at five sites internationally (mean prostate 59 cc). At 12 months, IPSS fell from 21.6 to 6.5 (-15.3), and Qmax rose from 10 to 20.8 mL/s. TRUS volume decreased 36% by month 3, ejaculatory function was largely preserved, and perioperative metrics were favorable (procedure 24 min; anesthesia 50 min). Early safety was acceptable overall, with transfusion in five patients (2.7%) [32].

Whiting et al. reported a single-center, prospective series of 55 men undergoing Aquablation with an athermal hemostasis strategy. At 12 months, prostate volume fell from 58 to 33 cc, Qmax rose from 9.9 to 23.9 mL/s, and IPSS improved from 21.7 to 6.1 and QoL from 4.8 to 1.4. Sexual and ejaculatory function were unchanged; Clavien ≥2 complications occurred in 14.5% of patients overall [33]. 

Desai et al. evaluated second-generation Aquablation in a prospective, single-center cohort of 47 men with symptomatic BPH. At three months, IPSS fell from 24.4 to 5.0, Qmax rose from 7.1 to 16.5 mL/s, and post-void residual decreased from 119 to 43 mL. The median hospital stay and catheterization were 3.1 and 1.9 days. Complications occurred in eight patients (five Clavien I/II, five III), confirming overall safety and effectiveness [34].

Comparative effectiveness 

Numerous interventional treatment approaches exist for BPH, encompassing a spectrum from non-ablative methods to resective procedures. Examples of resection techniques include open simple prostatectomy, which involves the surgical removal of the prostate's inner portion; laser enucleation, a precise method using laser energy to remove prostatic tissue; PVP, which utilizes a laser to vaporize obstructing tissue; and both monopolar and bipolar TURP, a common endoscopic procedure to excise excess prostatic tissue [11,33,35-37].

Functional Outcomes

A range of interventional treatments for BPH exists, including non-ablative and resective techniques. Resection techniques encompass open simple prostatectomy, laser enucleation, PVP, and TURP. While these various options prove effective in alleviating BPH symptoms, they are frequently associated with the unwelcome side effect of sexual dysfunction [11,33,35-37].

Aquablation has several advantages over traditional TURP, including comparable symptom improvement with less resection time, preservation of urinary continence, erectile function, and ejaculation, and minimized human errors due to robotic control, which ensures consistency and accuracy. Clinical trials like WATER and WATER II have shown that Aquablation offers similar or better functional outcomes than TURP, with a reduced risk of sexual dysfunction [24,34,38-41]. 

Gilling et al. compared Aquablation with TURP in a randomized trial and reported durable five-year outcomes. Symptom relief was sustained and similar overall (IPSS -15.1 vs. -13.2), while Aquablation showed greater benefit in prostates ≥50 mL (-3.5 points); objective flow gains persisted (Qmax +125% vs. +89%). Fewer patients required secondary BPH therapy after Aquablation (6% vs. 12.3%; ~51% lower). Ejaculatory function was better preserved and erectile function comparable, supporting Aquablation's superior long-term net benefit over TURP for men [42]. The authors also compared Aquablation with TURP in a blinded, randomized trial of 181 men with BPH, reporting two-year outcomes. Symptom relief (IPSS -14.7 vs. -14.9) and peak flow gains (11.2 vs. 8.6 mL/s) were similar. Sexual/ejaculatory function was preserved with Aquablation but declined slightly after TURP. Retreatment rates were low and comparable (4.3% vs. 1.5%) across multiple international centers [43].

Across studies of smaller prostates, including the phase II trial, WATER, and its US cohort, post-Aquablation hemoglobin fell by about 0.8-2 g/dL, with just one transfusion reported in WATER (1/116 ≈0.9%). In broader, less-selected series that treated larger glands (up to 118-154 mL; AquaBeam India and non-selected cohorts), the mean hemoglobin declines were ~1.4-1.78 g/dL, and transfusion rates were higher at 2.1-2.5%. Overall, bleeding burden is modest and rises slightly with larger prostate volumes [34,38,44].

Sexual Outcomes

In sexually active men, IIEF-15 scores remained stable after Aquablation but declined following TURP. Notably, the IIEF-15 overall sexual satisfaction subdomain favored Aquablation over TURP. Among 126 participants, the authors reported only one postoperative erectile dysfunction case in OPEN WATER, with a one-point SHIM decrease [32]. Consistently, other studies found no significant change in erectile function after Aquablation, regardless of prostate size [29,40,45,46].

Several studies have consistently demonstrated significantly better ejaculatory function in patients undergoing Aquablation compared to those treated with TURP [47,48]. This improved outcome in terms of ejaculatory preservation is a notable advantage of Aquablation in the management of BPH.

In a subgroup analysis of the American cohort of the WATER study, Kasivisvanathan and Hussain observed a significantly larger disparity in the anejaculation rate between the two groups at the one-year mark (9% vs. 45%; p=0.0006) [30]. At a one-year follow-up, Misrai et al. observed that 73.3% of patients maintained antegrade ejaculation [46]. The ability of surgeons to avoid the ejaculatory ducts and bladder neck, crucial for normal ejaculation, is attributed to the integration of image guidance and robotic assistance [49].

Learning Curve

Several studies highlighted the rapid learning curve of Aquablation, highlighting its efficiency and ease of adoption for medical professionals [38,46,50]. 

The current evidence suggests a relatively short acquisition time when compared to other available modalities. Previous publications have documented significantly longer learning curves for holmium laser enucleation of the prostate (HoLEP) (25-50 cases to attain proficiency), Greenlight (up to 100 cases to achieve proficiency), and TURP (reaching the performance plateau after 81 cases). This indicates a potentially faster path to surgeon competence with the newer method [51-54].

By contrast, a significant portion of surgeons participating in the WATER study (14 out of 17) had no prior experience with Aquablation, performing a median of only five cases throughout the duration of the trial. Similarly, surgeons involved in the WATER II trial possessed minimal previous experience, with a median of 0.5 procedures, and executed a median of four cases during the trial [45]. This highlights that many of the early studies on Aquablation involved surgeons who were still in the nascent stages of their learning curve with the technology.

Operative Time

Aquablation presents several notable advantages. Primarily, it boasts a remarkably short resection time, consistently achieving completion in under 10 minutes. This efficiency has been a consistent finding across numerous studies [55]. 

Bach et al. observed that the average operating room time for Aquablation decreased from 24.2 minutes for their initial 50 cases to 17 minutes for the subsequent 68 cases [38]. It is strongly believed that Aquablation's semi-automation would likely enhance reproducibility, reduce intraprocedural variability, and accelerate the learning curve [56].

Several studies have consistently demonstrated significantly faster resection and operative times in Aquablation procedures when compared to TURP [30,38,45,46,56]. This efficiency is a key advantage, potentially leading to reduced time under anesthesia and quicker recovery for patients.

Nguyen et al. analyzed operative times for various BPE procedures, finding that Aquablation took 30.1 minutes for prostate sizes between 30 and 80 mL and 40.7 minutes for prostate sizes between 80 and 150 mL [57].

**Figure 2:**
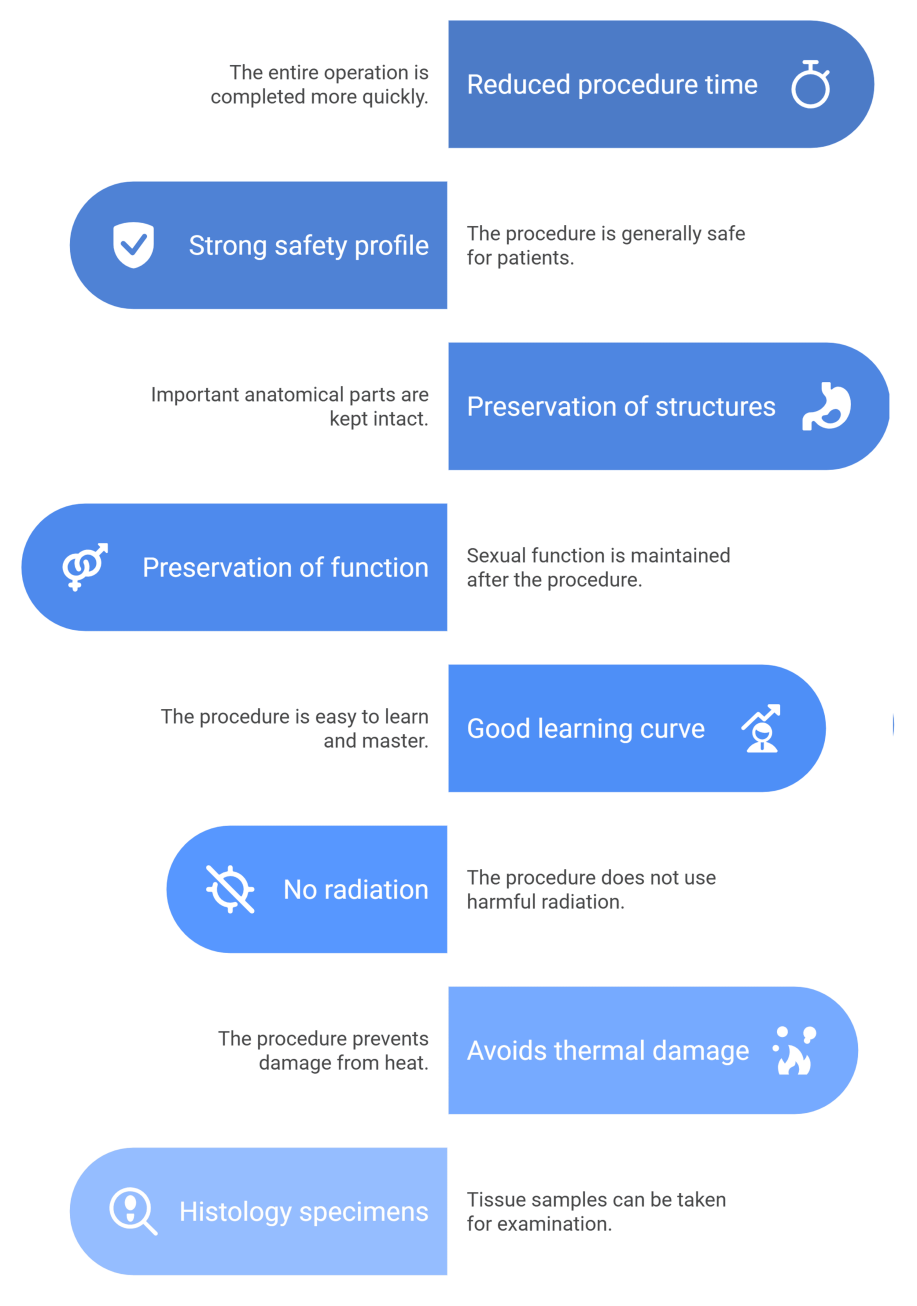
Advantages of the Aquablation for BPH BPH: benign prostatic hyperplasia The figure was created by the article authors to summarize the advantages of the Aquablation system using Canva (Canva Pty Ltd., Sydney, Australia).

Because lengthy resections can be dangerous, this high speed is crucial. Precise resection mapping and planning, guided by detailed radiography, enable the preservation of important anatomical features like the verumontanum and bladder neck (Figure 2) [58]. 

Limitations and future directions

While Aquablation offers several advantages, published complications include hemorrhage, as well as erectile and ejaculatory dysfunction. One previous study reported a high postoperative bleeding rate of 16.6%, which was likely due to limited surgical experience [10]. 

Aquablation's evidence base is collectively limited by modest sample sizes, single-center learning curve experiences, and relatively short follow-up in several reports. Eligibility in controlled trials was narrow (predominantly 30-80 mL prostates), while evidence for very large glands (80-150 mL) comes mainly from nonrandomized, single-arm cohorts, weakening causal inference. Even with five-year randomized data, outcomes show non-inferiority rather than clear superiority to TURP overall. Together, these factors constrain external validity, obscure rare or delayed adverse events, and temper confidence in broad, head-to-head generalizations across patient subgroups [31-34,42]. Outcomes evaluated in studies of robotic Aquablation for BPH include urinary flow rates, IPSS, procedure time, complication rates requiring retreatment, and preservation of sexual function. While some benefits like reduced operating room time, shorter hospital stays, day-case procedures, lower retreatment rates, and fewer adverse events have been claimed, more evidence is needed to support these. 

Aquablation appears promising for men prioritizing sexual function, but defining its long-term value requires stronger evidence. Future work should include multicenter randomized trials with at least 5-10 years of follow-up to quantify durability, retreatment rates, continence, and trajectories of erectile and ejaculatory function. Comparative effectiveness studies versus TURP, HoLEP, and PVP across prostate sizes and complex anatomies are needed, using standardized core outcomes (IPSS, Qmax, post-void residual) and validated sexual health instruments. Large real-world registries should capture safety, learning curve effects, and generalizability. Finally, cost-effectiveness analyses and optimization of patient selection, anticoagulation management, and same-day pathways will clarify system-level impact [23].

Beyond the growing interest in new surgical technologies, understanding the pathophysiology of BPH is crucial, particularly regarding metabolic syndrome. Patients with metabolic syndrome frequently exhibit prostatic inflammation, which is believed to be a potential cause of LUTS [59,60]. 

## Conclusions

Robotic Aquablation has emerged as a safe, effective, and durable surgical alternative for treating BPH, offering significant improvements in urinary outcomes that are comparable to the traditional TURP. The technology's main advantages are a superior sexual function preservation profile, with substantially lower rates of ejaculatory dysfunction, a rapid learning curve, and a consistently short resection time.

Despite these promising results, the existing evidence is limited by modest sample sizes and relatively short follow-up periods in some studies. Future research should prioritize multicenter randomized trials with long-term follow-up to definitively establish durability, retreatment rates, and comparative effectiveness against other BPH treatments like HoLEP and PVP.
